# Ultra-Weak Chemiluminescence Enhanced by Cerium-Doped LaF_3_ Nanoparticles: A Potential Nitrite Analysis Method

**DOI:** 10.3389/fchem.2020.00639

**Published:** 2020-08-07

**Authors:** Yufei Wang, Yanran Wang, Chunxia Huang, Tianyou Chen, Jing Wu

**Affiliations:** ^1^School of Science, China University of Geosciences, Beijing, China; ^2^Key Laboratory of Optic-electric Sensing and Analytical Chemistry for Life Science, MOE, Qingdao University of Science and Technology, Qingdao, China

**Keywords:** chemiluminescence, cerium, fluoride, nitrite, nanoparticles

## Abstract

In this work, cerium-doped LaF_3_ nanoparticles (LaF_3_:Ce NPs) were successfully synthesized and characterized. Its chemiluminescence (CL) property was studied, and it was amazingly found that it intensely enhanced the ultra-weak CL of the NaNO_2_-H_2_O_2_ system. The CL mechanism was systematically investigated and suggested to be the recombination of electron-injected and hole-injected LaF_3_:Ce NPs. The new CL system was developed to be a facile, original, and direct method for nitrite analysis. Experimental conditions were optimized and then a satisfactory linear relationship between CL intensity and nitrite concentration was obtained. This work introduced a new pathway for the research and application of traditional fluoride NPs doped with RE^3+^.

## Introduction

Fluoride is utilized as an ideal and appealing host for phosphors doped with rare earth ions (RE^3+^) owing to its adequate thermal and environmental stability as well as large solubility for RE^3+^ ions (Li et al., 2012). Compared with oxide systems, vibrational energies in fluorides is low and therefore trigger scarce quenching of the excited states of the RE^3+^ ions (Bender et al., 2000). Furthermore, RE^3+^-doped fluorides exhibit characteristic properties, such as high ionicity, low refractive index, wide band gap, and low phonon energy. KMgF_3_ (Schuyt and Williams, 2018), NaYF_4_ (Wu et al., 2019, 2020), NaGdF_4_ (Yi et al., 2019), and LaF_3_ (Bekah et al., 2016; Nampoothiri et al., 2017) have been investigated and exhibit high quantum yields and long luminescent lifetimes. RE^3+^-doped fluorides have been attracting attentions for several years due to the wide variety of technological applications including biomedical researches (All et al., 2019; Yan et al., 2019), biosensors (Vijayan et al., 2019), bioimaging (Hu et al., 2016; Han et al., 2017; Zeng et al., 2019), radiation detection (Ju et al., 2017), optoelectronic devices (Wu et al., 2018), and so on. However, to the best of our knowledge, the performance of RE^3+^-doped fluorides toward chemiluminescence (CL) has not been explored.

Nitrite is widely used in food manufacture as preservatives and fertilizing reagents. As an essential precursor of carcinogenic *N*-nitrosamine, excess intake of nitrite is harmful for human beings. Nitrite can cause irreversible conversion of hemoglobin to methemoglobin in the bloodstream and then bring detrimental effect for the oxygen transport in the whole body. In addition, nitrogen-based fertilizers and industrial wastewater pollute groundwater resources by nitrites. Thus, nitrite detection is of significant importance for food safety, public health, and environment protection (Wang et al., 2017; Zhang Y. et al., 2018; Cao et al., 2019). Various principle-based analytical methods have been devised for nitrite detection, such as electrochemical sensors (Ma et al., 2018; Wang et al., 2018; Zhou et al., 2019; Madhuvilakku et al., 2020), microplasma emission (Zheng et al., 2018), absorption spectrophotometry (Zhang L. et al., 2018), fluorescence (Dai et al., 2017; Jana et al., 2019; Pires et al., 2019), and CL (Lu et al., 2002, 2004; Lin et al., 2011; Wu et al., 2016). Electrodes are modified with complex strategies in electrochemical analysis. Special molecules need to be designed for spectrophotometric detections in order to amplify signal and reduce the background interferences. CL detections require simple instruments, interfere with low background, and are compatible with gas or aqueous phases. CL intensity was reported to be significantly enhanced by nanomaterials that gave promise for developing sensitive and convenient CL analytical methods. In 2011, carbon dots were firstly demonstrated to enhance the CL signal of the NaNO_2_-H_2_O_2_ system because of peroxynitrous acid generation (Lin et al., 2011). Nitrogen-rich quantum dots (QDs) were facilely synthesized and intensely enhanced the ultra-weak CL reaction of the NaIO_4_-H_2_O_2_ system through electron hole injection and CL resonance energy transfer (Zheng et al., 2017). In particular, molybdenum sulfide QDs were proved to give rise to the generation of reactive oxygen species from hydrogen peroxide (H_2_O_2_) in alkaline solution and gave a promise for CL emission (Dou et al., 2019). However, fluoride-based nanomaterials were scarcely utilized and the developed CL analysis was rarely applied in nitrite detection. Original CL detections for nitrites are worth giving research to pursue better performance.

In this work, cerium-doped LaF_3_ nanoparticles (LaF_3_:Ce NPs) were synthesized and firstly demonstrated to enhance the CL signal of the NaNO_2_-H_2_O_2_ system. Reactive oxygen species generation that was triggered by LaF_3_:Ce NPs was proved to be the main reason for CL enhancement. A linear relationship between the CL signal and nitrite concentration was found and implied that the LaF_3_:Ce NPs-NaNO_2_-H_2_O_2_ system could be applied in the determination of nitrite.

## Materials and Methods

### Reagents and Materials

Sodium nitrite (NaNO_2_) was purchased from Sinopharm Chemical Reagent Co., Ltd. (Shanghai, China). Sulfuric acid (H_2_SO_4_, 98%), H_2_O_2_ (35%), hydrochloric acid, and ethanol (98%) were brought from Beijing Chemical Reagent Co. (Beijing, China). Sodium fluoride (NaF, >98%), heptahydrate lanthanum chloride (LaCl_3_·7H_2_O, 99.9%), heptahydrate cerium chloride (CeCl_3_·7H_2_O, 99.9%), oleic acid (90%), 5,5-dimethyl-1-pyrroline *N*-oxide (DMPO), and ascorbic acid (AA) were all purchased from Sigma-Aldrich. Unless otherwise noted, all the chemicals were used without further purification.

### Apparatus

UV-vis absorption spectra were performed on a PerkinElmer Lambda 950 spectrophotometer. The photoluminescent (PL) spectra were collected on an Agilent Cary Eclipse spectrofluorometer. Fourier transform infrared (FT-IR) spectra were obtained on a PerkinElmer Frontier FT-IR spectrometer. CL experiments were conducted with an ultra-weak CL analyzer (IFFM-E, Xi'an Remex Analytical Instrument Co., Ltd, China). Transmission electron microscopy images were obtained on a JEOL-1400 transmission electron microscope (JEOL, Tokyo, Japan). Electron paramagnetic resonance (EPR) spectra were measured on a Bruker E500 spectrometer.

### LaF_3_:Ce NPs Synthesis

Hydrothermal reaction was utilized to synthesize LaF_3_:Ce NPs. 2.25 ml of LaCl_3_ solution (0.20 M), 1.00 ml of CeCl_3_ solution (0.05 M), 2.00 ml of NaF solution (1.00 M), 20 ml of ethanol, and 10 ml of oleic acid were mixed and stirred for 0.5 h in reaction kettle. The mixture was heated in an oven and kept at 200°C for 8 h. After reaction, the supernatant was removed. The remnant suspension was centrifuged at 6,000 rpm for 5 min and then the supernatant was also removed. The resultant solid was dispersed in 2.00 M hydrochloric acid. After ethanol addition, the mixture was centrifuged at 6,000 rpm for 5 min to remove the supernatant. The product was stored in 4 ml H_2_O for further use. The exact doping percentage of cerium was calculated to be 10%.

### CL Study of the LaF_3_:Ce NPs-NaNO_2_-H_2_O_2_ System

At first, CL intensities of the NaNO_2_-H_2_O_2_ system with and without LaF_3_:Ce NPs were compared. Fifty microliters of H_2_O_2_ (3.00 M), which was acidified by 0.04 M H_2_SO_4_, was injected into the mixture of 50 μl of LaF_3_:Ce NPs and 50 μl of NaNO_2_ (10 μM). In the control experiment, 50 μl of LaF_3_:Ce NPs was replaced by 50 μl of H_2_O. CL intensities of both the two conditions were recorded and compared. CL profiles were integrated at intervals of 0.1 s. Voltage of the photomultiplier tube (PMT) was set at 1.2 kV. CL spectrum was measured with high-energy cutoff filters (400–640 nm), which were set between the quartz cuvette and PMT as described in Cui et al. (2003). Additional orders of the reagents were investigated to collect CL kinetic curves. EPR measurements were operated at an X-band frequency of 9.85 GHz. Irradiation was performed by using a 300-W Xe lamp (300 nm < λ < 1,100 nm) with the output radiation focused on the samples in the cavity by an optical fiber (50 cm length, 0.3 cm diameter). All spectra were acquired at 298 K. DMPO (12.4 μl in 1 ml of H_2_O) was taken as the specific detection reagent for ·OH. AA (0.1 mM) was used as a scavenger for ${\text{O}}_{2}^{\cdot -}$. CL intensities of the LaF_3_:Ce NPs-NaNO_2_-H_2_O_2_ system with and without AA were recorded.

### Nitrite Analysis

Experimental conditions were optimized with different H_2_SO_4_ concentrations (0, 0.02, 0.03, 0.04, 0.05, and 0.06 M), H_2_O_2_ concentrations (0.00, 1.00, 2.00, 3.00, 4.00, and 5.00 M), and additional volumes of LaF_3_:Ce NPs (0, 10, 20, 30, 40, 50, 60, and 70 μl). The univariate method was adopted in systematically optimizing experimental parameters through changing one parameter at a time while keeping others constant. At the optimal experimental conditions, calibration curve was recorded by detecting CL intensities vs. different nitrite concentrations.

## Results

### Characterization of LaF_3_:Ce NPs

**Figure 1:**
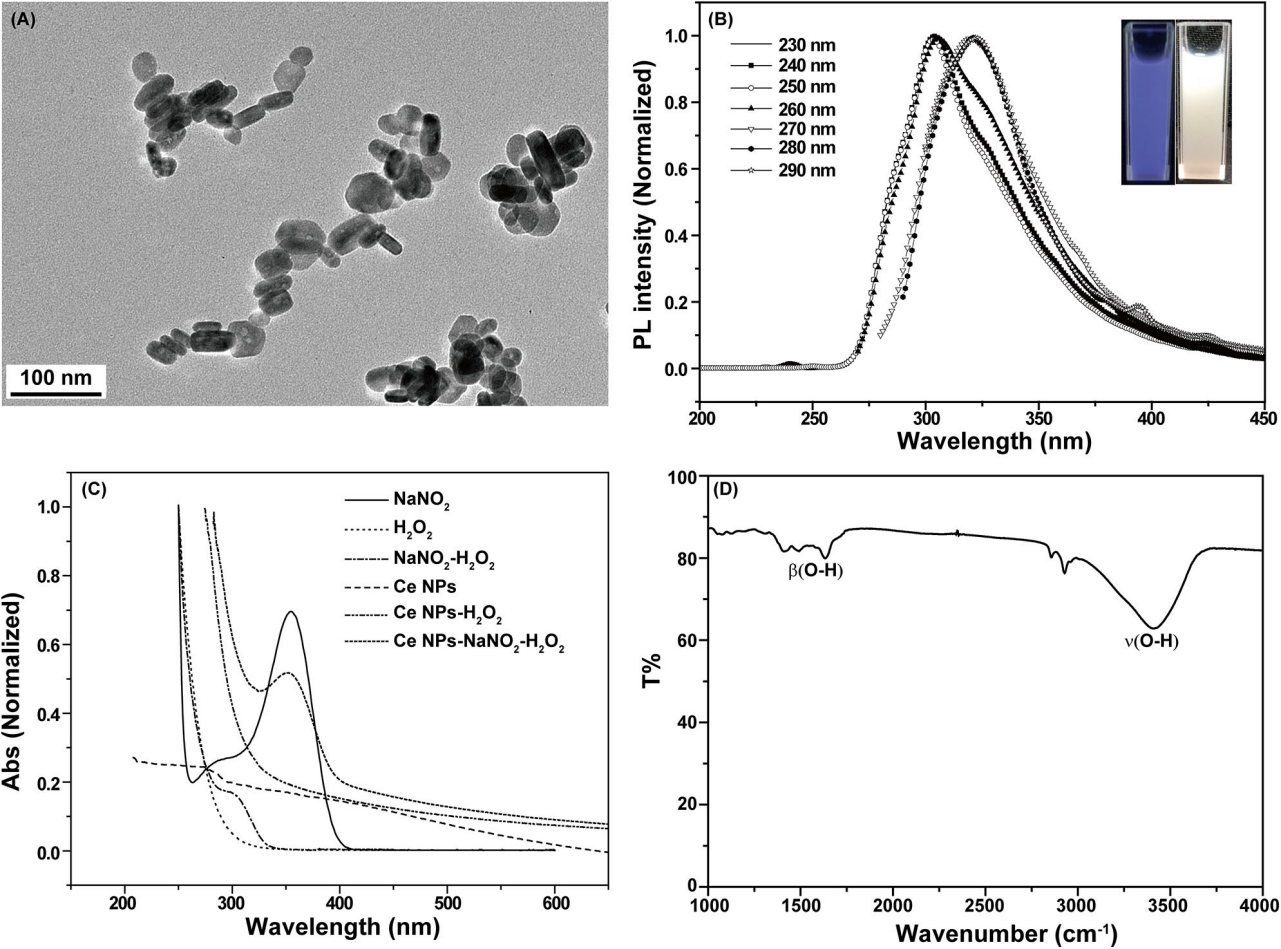
Characterization of LaF_3_:Ce NPs. **(A)** Transmission electron microscopy images of LaF_3_:Ce NPs. **(B)** PL spectra of LaF_3_:Ce NPs. The inset is the photograph of LaF_3_:Ce NPs under a UV lamp (λ_ex_ = 254 nm). **(C)** UV-vis absorption spectra of the reagent in the CL reaction. **(D)** FT-IR spectra of LaF_3_:Ce NPs.

LaF_3_:Ce NPs obtained in this work exhibited hexagonal phase and their average sizes were about 80 × 20 nm (Figure 1A). 4*f* shells of lanthanides are partially filled and are effectively shielded by outer 5s and 5p shells leading to satisfactory emissions. The prepared LaF_3_:Ce NPs gave a bright blue color under ultraviolet radiation (λ_ex_ = 254 nm) (Figure 1B, inset). The emission of LaF_3_:Ce NPs shifted to longer wavelength with the increase of excitation wavelength revealing the distribution of different surface energy traps of the LaF_3_:Ce NPs (Figure 1B). UV-vis absorption spectra of the LaF_3_:Ce NPs-NaNO_2_-H_2_O_2_ system were collected and are shown in Figure 1C. NaNO_2_ gave an absorption peak at 354 nm, which decreased when acidified H_2_O_2_ was added. Another absorption peak located at 301 nm appeared due to the isomerization of ONOOH, which was generated in the mixture of acidified H_2_O_2_ and NaNO_2_ (Lin et al., 2011), while no new absorption peaks were found when acidified H_2_O_2_ mixed with LaF_3_:Ce NPs. Except the absorption peak of ONOOH, no other new absorption peak was found in the LaF_3_:Ce NPs-NaNO_2_-H_2_O_2_ system, indicating that no new compound was formed. UV-vis absorption spectra gave some indications for the CL mechanism of this system, which was illustrated in detail in the subsequent section. FT-IR spectrum of LaF_3_:Ce NPs indicated that there were O-H groups on the surface of LaF_3_:Ce NPs (Figure 1D).

### CL of the LaF_3_:Ce NPs-NaNO_2_-H_2_O_2_ System

**Figure 2:**
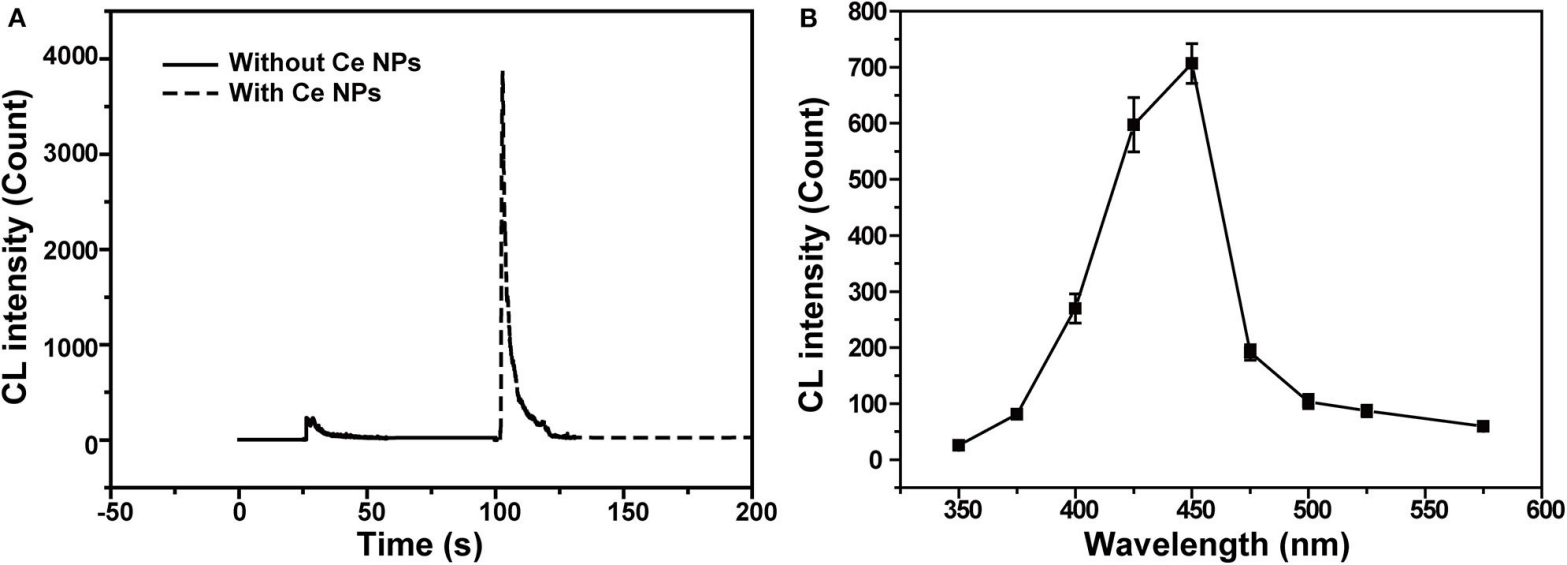
CL profiles of the LaF_3_:Ce NPs-NaNO_2_-H_2_O_2_ system. **(A)** Comparison of CL intensities of the NaNO_2_-H_2_O_2_ system with and without LaF_3_:Ce NPs. **(B)** CL spectrum of the LaF_3_:Ce NPs-NaNO_2_-H_2_O_2_ system. The standard error bars mean the variation of three individual experiments. Conditions: 3.00 M H_2_O_2_ in 0.04 M H_2_SO_4_, 50 μl of LaF_3_:Ce NPs and 10 μM NaNO_2_ solution. Voltage of the PMT was set at 1.2 kV.

CL intensities of the NaNO_2_-H_2_O_2_ system with and without LaF_3_:Ce NPs were sharply different. LaF_3_:Ce NPs addition intensely enhanced CL intensity (Figure 2A). As shown in Figure 2B, the CL spectrum for the LaF_3_:Ce NPs-NaNO_2_-H_2_O_2_ system was wide ranging from 375 to 500 nm and was centered at 450 nm. The fluorescent emission of LaF_3_:Ce NPs is also wide, which is similar to the CL spectrum of the LaF_3_:Ce NPs-NaNO_2_-H_2_O_2_ system. As a result, it is reasonable to refer that the CL originates from the various surface energy traps existing on the LaF_3_:Ce NPs. Compared with the PL peak of LaF_3_:Ce NPs, the CL spectrum is red-shifted due to the energy separations of LaF_3_:Ce NPs surface states. PL was generated through excitation and emission within the core of the LaF_3_:Ce NPs and the energy gap between them is larger than the energy separations on NPs surface (Ding et al., 2002; Myung et al., 2002).

### CL Kinetic Study

**Figure 3:**
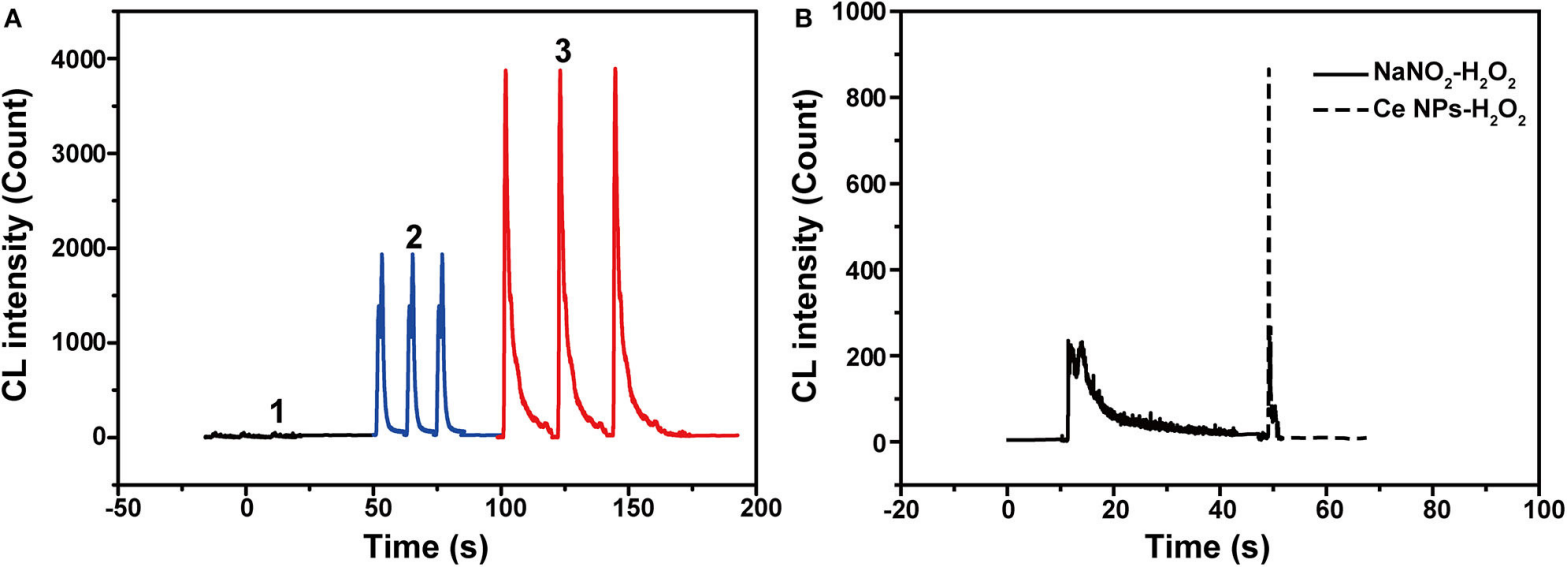
CL kinetic study of the LaF_3_:Ce NPs-NaNO_2_-H_2_O_2_ system. **(A)** CL kinetic curves of the LaF_3_:Ce NPs-NaNO_2_-H_2_O_2_ system with different reagent mixing orders: **1**. injecting LaF_3_:Ce NPs into the mixture of NaNO_2_ and acidified H_2_O_2_; **2**. injecting NaNO_2_ into the mixture of LaF_3_:Ce NPs and acidified H_2_O_2_; **3**. injecting acidified H_2_O_2_ into the mixture of LaF_3_:Ce NPs and NaNO_2_. CL signals of three repeated experiments were given. **(B)** CL kinetic curves of the NaNO_2_-H_2_O_2_ system and the LaF_3_:Ce NPs-H_2_O_2_ system. Conditions: 3.00 M H_2_O_2_ in 0.04 M H_2_SO_4_, 50 μl of LaF_3_:Ce NPs and 10 μM NaNO_2_ solution. Voltage of the PMT was set at 1.2 kV.

As described in UV-vis absorption spectra, ONOOH was generated when NaNO_2_ was mixed with acidified H_2_O_2_ (Equation 1) (Anbar and Taube, 1954). ONOOH easily transforms to be nitrate via the stage of HOONO^*^ and give emissions during the process (Equation 2) (Houk et al., 1996). The emission locates at 350–450 nm, which overlaps the absorption spectrum of LaF_3_:Ce NPs. Hence, LaF_3_:Ce NPs can be excited by the energy of transformation and cause CL emission. However, the maximum of the transformation-derived CL was obtained at the pH value of 6.5–7.0 while the maximum CL of the LaF_3_:Ce NPs-NaNO_2_-H_2_O_2_ system was recorded in a severe acidic solution (Starodubtseva et al., 1999). As a consequence, the transformation energy only partially contributed to the CL of the LaF_3_:Ce NPs-NaNO_2_-H_2_O_2_ system. Various mixing orders of reagents influenced the reactions between LaF_3_:Ce NPs and ONOOH and then affected the CL intensities (Figure 3A). The highest CL was obtained when acidified H_2_O_2_ was injected into the mixture of LaF_3_:Ce NPs and NaNO_2_. At this condition, the generated ONOOH adequately reacted with LaF_3_:Ce NPs and gave enhanced CL. Mixing of NaNO_2_ with acidified H_2_O_2_ without LaF_3_:Ce NPs gave weak and lasting CL while mixing of LaF_3_:Ce NPs with acidified H_2_O_2_ without NaNO_2_ gave weak and rapid CL (Figure 3B).

(1) $$HNO_{2}+H_{2}O_{2}\to ONOOH+H_{2}O$$

(2) $$ONOOH\to ONOOH^{*}\to NO_{3}^{-}+H^{+}$$

### CL Mechanism

**Figure 4:**
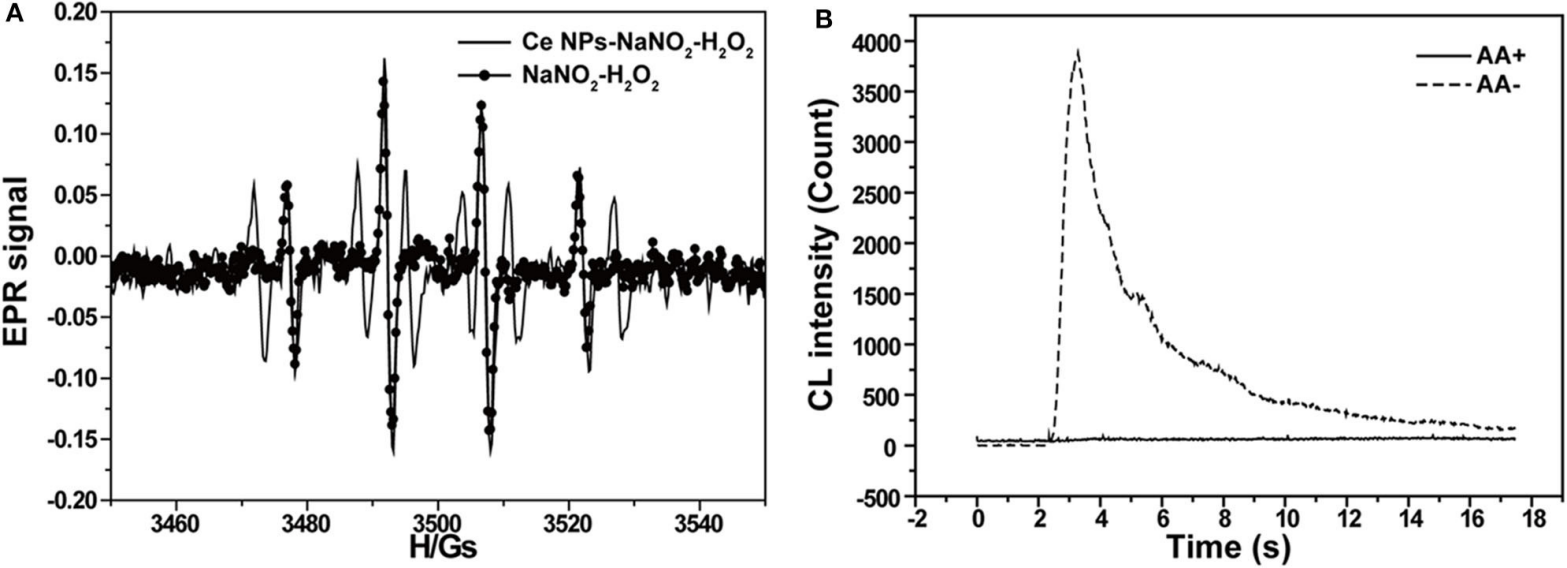
CL mechanism study of the LaF_3_:Ce NPs-NaNO_2_-H_2_O_2_ system. **(A)** EPR spectra of ·OH generated via the reaction of DMPO probe in the LaF_3_:Ce NPs-NaNO_2_-H_2_O_2_ and NaNO_2_-H_2_O_2_ systems. **(B)** CL profiles of the LaF_3_:Ce NPs-NaNO_2_-H_2_O_2_ system with and without AA. Conditions: 3.00 M H_2_O_2_ in 0.04 M H_2_SO_4_, 50 μl of LaF_3_:Ce NPs, 10 μM NaNO_2_ solution, 12.4 μl of DMPO in 1 ml of H_2_O, and 0.1 mM AA. Voltage of the PMT was set at 1.2 kV.

**Figure 5:**
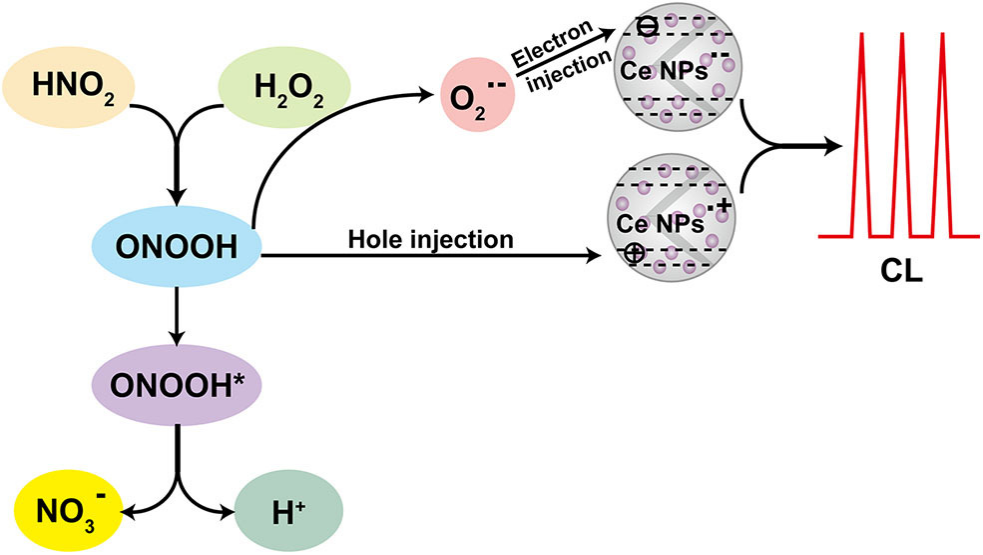
Schematic illustration of the CL mechanism of the LaF_3_:Ce NPs-NaNO_2_-H_2_O_2_ system.

According to the CL kinetic study, it demonstrated that the reactions between LaF_3_:Ce NPs and ONOOH or its related species were the main cause accounting for the enhanced CL. ONOOH was reported to be capable of producing reactive oxygen species (Equations 3–5) (Alvarez et al., 1995; Gunaydin and Houk, 2008; Lin et al., 2011). It was obvious that ONOOH-produced reactive oxygen species include ·OH, ${\text{O}}_{2}^{\cdot -}$, and ^1^O_2_ in this system. EPR was performed and DMPO was utilized as the specific detection reagent for ·OH to directly examine the variation of ·OH after LaF_3_:Ce NPs addition. Although CL intensity of the LaF_3_:Ce NPs-NaNO_2_-H_2_O_2_ system was greatly enhanced, the production of ·OH was almost not increased (Figure 4A). ^1^O_2_ was derived from ·OH so it could refer that there was no increase in ^1^O_2_ quantity. Ethanol was reported to react with ·OH and yield an octet spectrum that was completely distinct from the DMPO-OH spectrum (Finkelstein et al., 1980). The octet spectrum in the LaF_3_:Ce NPs-NaNO_2_-H_2_O_2_ system rooted in the reaction between ·OH and residual ethanol from treatment process of LaF_3_:Ce NPs. Furthermore, AA, which was a scavenger for ${\text{O}}_{2}^{\cdot -}$, obviously inhibited the CL of the LaF_3_:Ce NPs-NaNO_2_-H_2_O_2_ system (Figure 4B). All the results indicated that ${\text{O}}_{2}^{\cdot -}$ was the critical reason for the enhanced CL instead of ·OH and ^1^O_2_. ${\text{O}}_{2}^{\cdot -}$ acting as an electron donor reacted with LaF_3_:Ce NPs to produce LaF_3_:Ce NPs^·−^ (Equation 6) (Poznyak et al., 2004). ONOOH serving as a hole injector converted LaF_3_:Ce NPs to LaF_3_:Ce NPs^·+^ (Equation 7). Electron–hole annihilation between LaF_3_:Ce NPs^·−^ and LaF_3_:Ce NPs^·+^ resulted in CL emission (Equation 8) (Figure 5; Ding et al., 2002; Poznyak et al., 2004; Zheng et al., 2009; Dong et al., 2010).

(3) $$ONOOH\to \cdot NO_{2}+\cdot OH$$

(4) $$ONOOH+H_{2}O_{2}\to O_{2}^{\cdot -}+\cdot NO_{2}+H^{+}+H_{2}O$$

(5) O2·−+·OH→O12+OH−

(6) $$LaF_{3}:CeNPs+O_{2}^{\cdot -}\to LaF_{3}:CeNPs^{\cdot -}+O_{2}$$

(7) $$LaF_{3}:CeNPs+ONOOH\to LaF_{3}:CeNPs^{\cdot +}+\cdot NO_{2}+H_{2}O$$

(8) $$\begin{array}{c} LaF_{3}:CeNPs^{\cdot +}+LaF_{3}:CeNPs^{\cdot -}\to LaF_{3}:CeNPs^{*}\\ +LaF_{3}:CeNPs\to 2LaF_{3}:CeNPs+hv \end{array}$$

### Nitrite Analysis

**Figure 6:**
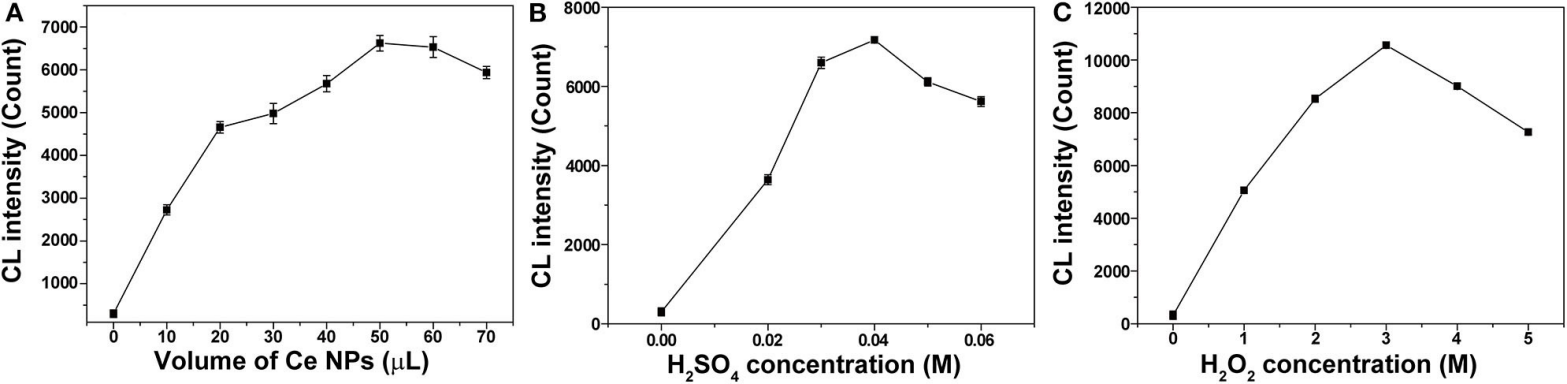
Optimization of experimental conditions for nitrite analysis. **(A)** Volume of LaF_3_:Ce NPs added into the CL system was optimized. **(B)** Concentration of H_2_SO_4_ used to acidify H_2_O_2_ was optimized. **(C)** Concentration of H_2_O_2_ was optimized. The standard error bars mean the variation of three individual experiments.

To establish the optimal conditions for nitrite analysis, the volume of LaF_3_:Ce NPs added into the CL system and concentrations of H_2_SO_4_ and H_2_O_2_ were investigated, respectively. As shown in Figure 6A, 50 μl of LaF_3_:Ce NPs was added into the CL system and provided the highest CL emission. Less LaF_3_:Ce NPs inadequately reacted with ONOOH while surplus LaF_3_:Ce NPs also consumed energy. Reactive substance ONOOH was the product of NaNO_2_ and H_2_O_2_ in acid medium, so H_2_SO_4_ was indispensable for the CL system. No CL signals could be observed in the absence of H_2_SO_4_. The most intense CL signal was obtained with the H_2_SO_4_ concentration of 0.04 M (Figure 6B). CL signal increased with the concentration of H_2_O_2_ in the range from 0 to 3.00 M (Figure 6C). Hence, the optimal analytical conditions for nitrite analysis were 3.00 M H_2_O_2_ in 0.04 M H_2_SO_4_ injected into the mixture of 50 μl of LaF_3_:Ce NPs and nitrite solution.

**Figure 7:**
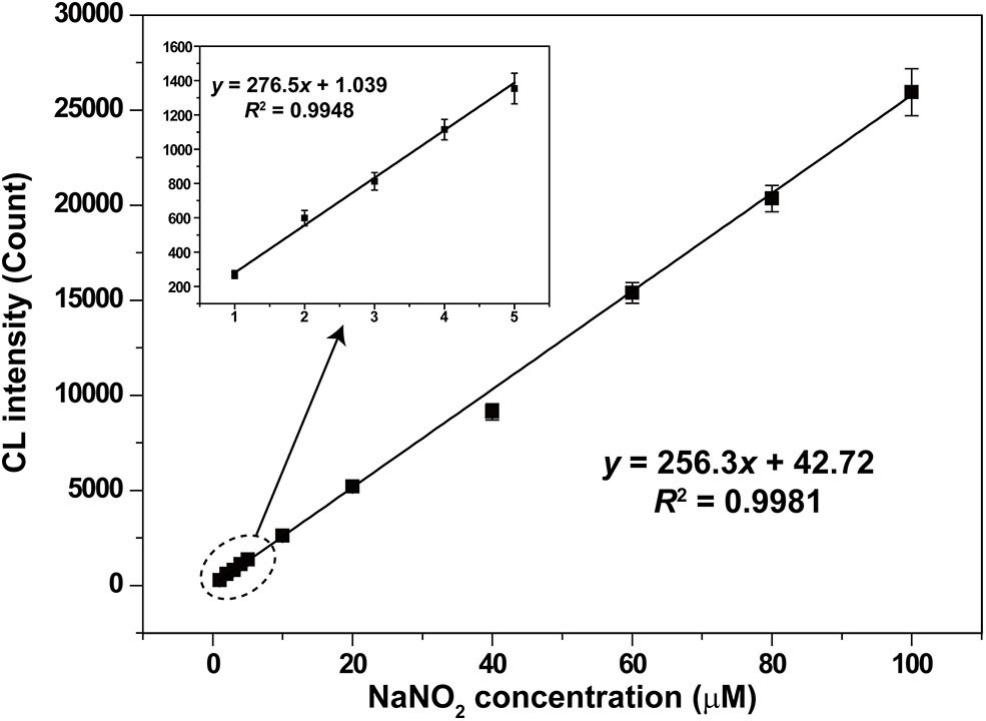
Calibration curve for nitrite analysis based on the LaF_3_:Ce NPs-enhanced CL. The standard error bars mean the variation of three individual experiments. Conditions: 3.00 M H_2_O_2_ in 0.04 M H_2_SO_4_ and 50 μl of LaF_3_:Ce NPs. Voltage of the PMT was set at 1.2 kV.

Under the optimal conditions, CL signals for different nitrite concentrations were recorded and shown in Figure 7. Good linear relationship between CL intensity and nitrite concentration was obtained in the range from 1 to 100 μM with a correlation coefficient of 0.9981 (*y* = 256.3*x* + 42.72). The relative standard deviation values of the analysis were 8.7, 1.2, and 4.8% for nitrite concentrations of 1, 10, and 100 μM, respectively. Relative standard deviation values demonstrated the satisfactory reproducibility. The limit of detection (*S*/*N* = 3) for nitrite was 0.33 μM.

## Discussion

The eternal goals and challenges of analytical chemistry are developing accurate, automated, selective, stable, sensitive, high-speed, high-throughput, and *in situ* analytical methods and protocols (Ju, 2013). The combination of analytical chemistry with new materials, especially nanomaterials, is the current frontier research topics and exhibits greatly improved analytical capacities. CL analysis is a traditional analytical technology and possesses outstanding advantages, such as low cost, simple instrument, fast response, and high compatibility. The application of nanomaterials in CL analysis leads to new CL sensing disciplines and offers a broad palette of opportunities for analytical chemists. In 2004, Poznyak et al. (2004) firstly reported the nanocrystal band gap CL derived from CdSe/CdS core-shell QDs that acted as a novel class of luminophores with the emission state originated from quantum-confined orbitals. Superior emission properties in QDs gave promises for developing QD-based nanoprobes for CL analysis. Besides traditional semiconductor QDs, some novel nanomaterials, such as carbon nanodots (Lin et al., 2011), graphene QDs (Hassanzadeh and Khataee, 2018), graphitic carbon nitride QDs (Zhu et al., 2019), and N-dots (Zheng et al., 2017), were developed to be potential platforms for CL sensing. These nanomaterials are superior in terms of robust chemical inertness, low toxicity, good aqueous solubility, high resistance to photobleaching, and satisfactory biocompatibility. Our work is an endeavor step during the development process of nanomaterial-sensitized CL analysis methods. In this study, LaF_3_:Ce NPs were successfully synthesized and applied in nitrite detection based on CL signals. The synthetic process of LaF_3_:Ce NPs was simple and the products were fully characterized to give indications for the CL mechanism study. The enhancement of LaF_3_:Ce NPs for the NaNO_2_-H_2_O_2_ CL system was efficient and the mechanism was systematically and scientifically explained. The linear relationship between CL intensity and nitrite concentration was found, although there were spaces for improving the limit of detection. This work tried to explore new CL nanoprobes and gave a new route for fluoride applications. In the future, there is still a great demand for developing novel CL nanoprobes especially metal-free QDs and two-dimensional QDs.

## Conclusions

In summary, LaF_3_:Ce NPs were successfully synthesized and demonstrated to intensely enhance ultra-weak CL of the NaNO_2_-H_2_O_2_ system. The CL mechanism was suggested to be the electron–hole annihilation between hole-injected and electron-injected LaF_3_:Ce NPs. The new CL system was developed to be a novel, simple, and straightforward analytical method for nitrite. All the experimental conditions were optimized and a satisfactory linear relationship between CL intensity and nitrite concentration was obtained. This work shed a new light on the research and application of traditional fluoride NPs doped with RE^3+^.

## Data Availability Statement

The raw data supporting the conclusions of this article will be made available by the authors, without undue reservation, to any qualified researcher.

## Author Contributions

YuW organized and conducted all the experiments, analyzed data, and wrote the manuscript. JW coordinated the project, supervised all the experiments, analyzed data, and wrote, edited, and reviewed the manuscript. YaW assisted the experiments of nitrite analysis. CH synthesized and characterized LaF_3_:Ce NPs. TC performed experiments of the CL study of the LaF_3_:Ce NPs-NaNO_2_-H_2_O_2_ system. All authors contributed to the article and approved the submitted version.

## Conflict of Interest

The authors declare that the research was conducted in the absence of any commercial or financial relationships that could be construed as a potential conflict of interest.

## Abbreviations

AA: ascorbic acid
LaF_3_:Ce NPs: cerium-doped LaF_3_ nanoparticles
CL: chemiluminescence
EPR: electron paramagnetic resonance
FT-IR: Fourier transform infrared
PL: photoluminescent
PMT: photomultiplier tube
QDs: quantum dots
RE^3+^: rare earth ions.
